# THE AGING BRAIN AND MOTOR LEARNING

**DOI:** 10.1093/geroni/igz038.2430

**Published:** 2019-11-08

**Authors:** Qu Tian, Roger Mullins, Abby Corkum, David Reiter, Daniel Pupo, Eleanor M Simonsick, Dimitrios Kapogiannis, Stephanie Studenski

**Affiliations:** 1 National Institute on Aging, Baltimore, Maryland, United States; 2 Emory University, Atlanta, Georgia, United States; 3 Johns Hopkins Bloomberg School of Public Health, Baltimore, Maryland, United States; 4 Longitudinal Studies Section, Intramural Research Program, National Institute on Aging, Baltimore, Maryland, United States; 5 University of Pittsburgh, Pittsburgh, Pennsylvania, United States

## Abstract

The effect of aging on motor learning is poorly understood. This study investigated response time and patterns of brain activation induced over the course of a bimanual motor learning task in three age groups. Twenty-two cognitively unimpaired participants (32%women) were grouped into Young (<35,n=6), Middle-Age (36-59,n=10), and Old (60+,n=6). A self-paced bimanual motor learning task was performed during fMRI. The task consisted of using 2 capital and 2 lower case letters in strings of 16 cues with 6 novel alternating with 6 repeated sequence blocks. To assess learning, a repeated measures ANOVA tested whether average time per slide differed over time between novel and sequence conditions. Voxel-wise changes in brain activation between novel and sequence conditions over time were examined using a within-subject repeated measures model. Faster initial time per slide was associated with younger age (p0.05). Old had increased brain activation in repeated sequence than novel conditions in right postcentral and superior parietal regions during the early half of the task compared to the second half (p0.05). We found behavioral evidence of motor learning in Middle-Age and Old, but not Young, perhaps because younger individuals performed quickly and learned sequence almost immediately. Among older individuals, sequence-specific learning in parietal regions challenges the view that it is mediated by only motor areas.

